# Borderline personality symptoms and work performance: a population-based survey

**DOI:** 10.1186/s12888-018-1777-9

**Published:** 2018-06-19

**Authors:** Trees T. Juurlink, Margreet ten Have, Femke Lamers, Hein J. F. van Marle, Johannes R. Anema, Ron de Graaf, Aartjan T. F. Beekman

**Affiliations:** 1 Department of Psychiatry, VU University Medical Centre, Amsterdam Public Health Research Institute, Oldenaller 1, 1081 HJ Amsterdam, The Netherlands; 2 Netherlands Institute of Mental Health and Addiction, Trimbos Institute, Utrecht, The Netherlands; 3 Department of Social Medicine, VU University Medical Centre, Amsterdam Public Health Research Institute, Amsterdam, The Netherlands

**Keywords:** Borderline personality symptoms, Public health, Employment, Work performance, Occupational health

## Abstract

**Background:**

This study aims to elucidate the interplay between borderline personality symptoms and working conditions as a pathway for impaired work performance among workers in the general population.

**Methods:**

Cross-sectional data from the Netherlands Mental Health Survey and Incidence Study-2 (NEMESIS-2) were used, including 3672 workers. Borderline personality symptoms were measured with the International Personality Disorder Examination (IPDE) questionnaire. Working conditions (decision latitude, psychological job demands, job security and co-worker support) were assessed with the Job Content Questionnaire (JCQ). Impaired work performance was assessed as total work loss days per month, defined as the sum of days of three types of impaired work performance (inability to work, cut-down to work, and diminished quality at work). These were assessed with the WHO Disability Assessment Schedule (WHO-DAS). Common mental disorders (CMD) were assessed with the Composite International Diagnostic Interview (CIDI).

**Results:**

Number of borderline personality symptoms was consistently associated with impaired work performance, even after controlling for type or number of adverse working conditions and co-occurrence of CMD. Borderline personality symptoms were associated with low decision latitude, job insecurity and low co-worker support. The relationship between borderline personality symptoms and work performance diminished slightly after controlling for type or number of working conditions.

**Conclusions:**

The current study shows that having borderline personality symptoms is a unique determinant of work performance. This association seems partially explained through the impact of borderline personality symptoms on working conditions. Future studies are warranted to study causality and should aim at diminishing borderline personality symptoms and coping with working conditions.

## Background

Borderline personality disorder (BPD) is a severe mental disorder characterized by impulsivity, emotional instability, interpersonal dysfunction, perturbed self-image and severe functional impairment [1, 2]. BPD is associated with unemployment, extensive use of social benefits, and therefore high societal costs [2–4]. Ten Have and colleagues [5] found that even minimal borderline personality symptoms are associated with functional impairment and unemployment. Furthermore, Zimmerman and colleagues [6] found that individuals with one borderline personality symptom had significantly more common mental disorders (CMD), psychiatric hospitalizations and missed time from work compared to individuals with no borderline personality symptoms. Extensive, research emphasizes that BPD should be studied as a dimensional construct, because BPD is heterogeneous and trait severity differs [7, 9]. However, little is known about the prevalence of borderline personality symptoms and functioning among those still at work. Studying risk factors for impaired work performance is important, because the costs due to work loss constitute the bulk of total societal costs associated with mental disorders [6]. Furthermore, most people want to work, emphasizing the importance for interventions aimed at improving work performance.

**Fig. 1:**
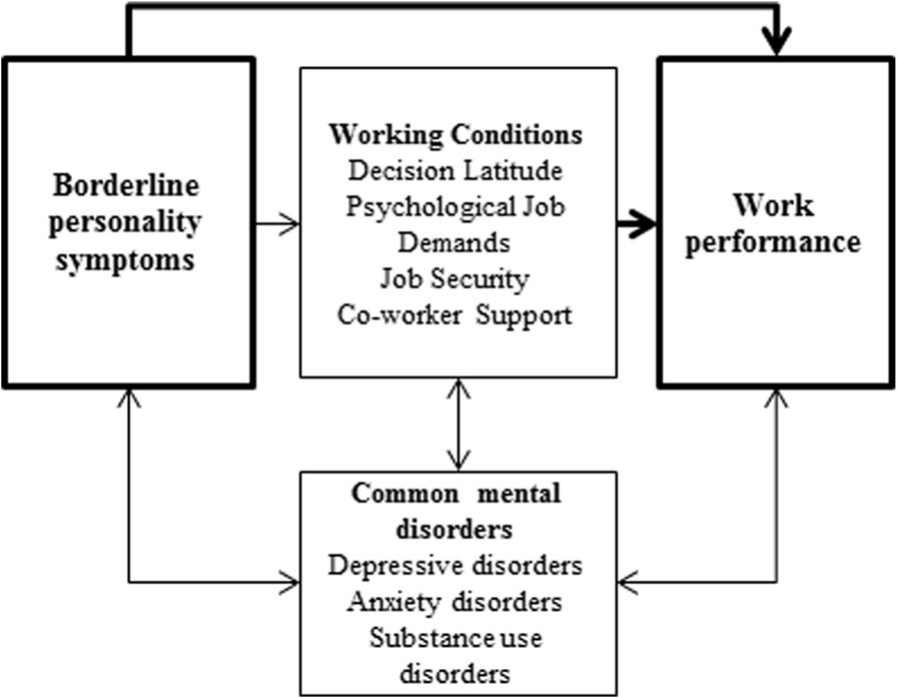
Proposed model of the interplay between borderline personality symptoms, working conditions and concurrent common mental disorders as a pathway for work performance. Thick arrows indicate direct effect and thin arrows indirect effect. Bidirectional arrows indicate potential confounding variables

Impaired work performance is often defined as absenteeism (days a worker is absent) and presenteeism (days of reduced functioning while at work) [7]. Potential risk factors of impaired work performance are mental health, such as common mental disorders and personality disorders [2, 7, 8], and adverse working conditions [9]. The job demands-control model of Karasek is often used for measuring psychosocial working conditions such as decision latitude, psychological job demands, job security and co-worker support [10]. Plaisier and colleagues [11] showed that low co-worker support and low decision latitude were associated with higher absenteeism among workers with and without depressive and anxiety disorders. Vlasveld and colleagues [12] showed that personality characteristics are associated with absenteeism in both healthy workers and workers with depressive and anxiety disorders. We expect that this is also true for workers with borderline personality symptoms and therefore hypothesize that borderline personality symptoms influence work performance and that adverse working conditions will mediate the relationship between borderline personality symptoms and impaired work performance (Fig. 1).

With respect to the working conditions, we expect (i) that borderline personality symptomatology diminishes the experienced decision latitude because individuals with BPD have been shown to experience difficulties in planning, decision-making and controlling their impulses [13, 14]. Difficulties with planning and decision-making might increase feelings of stress. Thus, we hypothesize (ii) that workers with borderline personality symptoms experience high psychological job demands. Individuals with BPD were previously found at increased risk for dismissal and demotion [2] and therefore we anticipate (iii) that workers with borderline personality symptoms experience high job insecurity. Interpersonal dysfunction is a key feature of BPD [15] which could lead to conflicts in the workplace [2, 4]. Consequently, we expect (iiii) that workers with borderline personality symptoms will experience low co-worker support.

Borderline personality symptoms often co-occur with common mental disorders (CMD), such as depression and anxiety [5]. These are by themselves associated with absenteeism [16, 17] and presenteeism [7]. Therefore it is important to control for concurrent CMD when studying the interplay between borderline personality symptoms, working conditions on work performance. We used a community based sample from the Netherlands Mental Health Survey and Incidence Study-2 (NEMESIS-2) and aimed to test (i) the association between borderline personality symptoms and impaired work performance, (ii) whether this association was mediated by adverse working conditions and which working conditions are associated with borderline personality symptoms, while (iii) taking the effect of concurrent CMD into account.

## Methods

### Sample

Data were used from the second wave of NEMESIS-2, in which borderline personality symptoms were assessed and questionnaires on working conditions and work performance were administered. For the present study we selected 3672 participants (1831 men and 1841 women) with a paid job of > 12 h per week (as in: Ten Have et al. [18]).

NEMESIS-2 is a nationally representative survey of the general adult population in the Netherlands aged 18 to 64 years [5, 19]. Participants were selected from households based on multistage, stratified random sampling, selecting one participant per household. In the first wave (T0) from November 2007 to July 2009, a total of 6646 persons were interviewed (response rate 65.1%; average interview duration: 95 min). Although younger participants were slightly underrepresented, the total sample was nationally representative. Interviews were generally held at the participant’s home and all interviews were computer-assisted. Three years after T0 from November 2010 to June 2012, participants were approached for follow-up (T1). In this second wave 5303 persons were re-interviewed (response rate 80.4%, those deceased excluded; average interview duration: 84 min). Attrition rate was not significantly associated with common mental disorders at baseline, after adjusting for sociodemographic characteristics [20]. For rationale, objectives and methods of NEMESIS-2 see De Graaf and colleagues [19]. The NEMESIS-2 study protocol was approved by a medical ethics committee, and all participants provided written informed consent.

### Measures

Borderline personality symptoms were measured using eight questions from the International Personality Disorder Examination (IPDE) [18] corresponding with the DSM symptom criteria for BPD [21]. These questions are part of the Composite International Diagnostic Interview (CIDI) 3.0 – a fully structured lay-administered diagnostic interview [22]. Each question of the IPDE resembles a criterion for BPD [23]. A true-false inventory format was used and the accumulative scores of the total sum of ‘true’ responses were assessed. The higher the score, the larger the number of borderline personality symptoms. Internal consistency was poor (α = 0.53), however this is explained by the variability of the items. The IPDE does not assess one criterion of BPD (recurrent suicidal behaviour, gestures or threats, or self-mutilating behaviour). In a subsample of the National Comorbidity Survey Replication (NCS-R) in the United States, performing a clinical reappraisal interview, the IPDE was found valid for the assessment of BPD [24].

Working conditions were assessed with the Job Content Questionnaire (JCQ) [25]. Four working conditions were used: decision latitude (9 items, α = 0.81), psychological job demands (5 items, α = 0.60), job security (3 items, α = 0.67) and co-worker support (4 items, α = 0.79). Response categories were based on 4-point Likert scales ranging from ‘strongly disagree’ to ‘strongly agree’, except for two questions on job security that were based on 3-point Likert scales. The number of missing values on each scale was very small, except for co-worker support (9.1%) where the missing values were almost all due to workers without colleagues. We kept these missing values and did not redefine them as having no adverse working condition. With respect to borderline personality symptoms, workers without colleagues were not significantly differing in number of borderline personality symptoms compared to those with low or high co-worker support.

Additionally, the number of adverse working conditions was assessed as a measure of job quality consistent with previous studies [17, 24]. The adverse working conditions were first defined as present on each scale if a score fell in the quartile of the distribution that corresponded to the greatest adversity (e.g. low latitude, high demands, low security and low support). The four adversities were then summed to report the experienced number of adverse working conditions. Missing values on any of the separate working condition adversities, except for low co-worker support, resulted in a missing on the summary measure of number of adverse working conditions (1.1%). The measure ranged from 0 to 3 or more adversities and was analysed as an ordinal variable.

Work performance was conceptualized as absenteeism and presenteeism and assessed by three questions on the WHO Disability Assessment Schedule (WHO-DAS) [26]. The questions related to impaired work performance due to illness of the past 30 days and specifically asked the following: (a) “How many days out of the past 30 were you totally unable to work or carry out your normal activities?”, (b) “How many days out of the past 30 were you able to work and carry out normal activities, but had to cut down on what you did or not get as much done as usual?” and (c) “How many days out of the past 30 did you cut back on the quality of your work or how carefully you worked?”. Total work loss days were based on the sum of days of the three different types of work loss, as previously published [7]. In case of absence for all working days, the two answers on reduced functioning were assigned a value of zero. One day of reduced functioning was counted as half in line with other studies [24, 27]. The maximum number of lost workdays was set at 21.5 days per month for fulltime workers and proportioned for part-time workers. The following categories were used for analysis: 0, 0.1–5 or > 5.1 days of work loss.

Presence of CMD was assessed with the CIDI version 3.0, which was developed and adapted for use in the World Mental Health Survey Initiative [22]. An improvement on the Dutch version of the CIDI 3.0 was used in NEMESIS-2. The 12-month disorders include: mood disorder (i.e. major depression, dysthymia and bipolar disorder), anxiety disorders (i.e. panic disorder, agoraphobia, social phobia, specific phobia and generalized anxiety disorder) and substance use disorders (alcohol/drug abuse and dependence). The CIDI 3.0 was found to assess mood, anxiety and substance use disorders with generally good validity in comparison to blinded clinical reappraisal interviews [28].

Next to mood, anxiety and substance use disorders, sex, age, education, and living situation (with or without partner) were considered putative confounders, since these variables are associated with BPD [5]. Mood, anxiety and substance use disorders are furthermore associated with working conditions and work performance [11, 17].

### Statistical analyses

All analyses were performed with STATA version 12.1, using weighted data to correct for differences in the response rates in several sociodemographic groups at both waves and differences in the probability of selection of respondents within households at baseline. Robust standard errors were calculated in order to obtain correct 95% confidence intervals and *p*-values [29].

First, the presence of four categories of number of borderline personality symptoms among this working population were calculated (0, 1–2, 3–4, and ≥5 symptoms). People with ≥5 borderline personality symptoms can be viewed as suffering from BPD, since they fulfil the required number of DSM-IV criteria (at least 5 out of 9) for a BPD diagnosis [30].

Second, the mean number of borderline personality symptoms in sociodemographic characteristics and 12-months common mental disorders were calculated using simple descriptive analyses to study potential confounders.

Third, multivariate linear and multinomial logistic regression analyses were performed to study the association between borderline personality symptoms and type and number of adverse working conditions. In the first series of analyses, adjustments were made for sex and age. In the second series of analyses, additional adjustments were made for education, living situation, any 12-month mood disorder, any 12-month anxiety disorder, and any 12-month substance use disorder.

Fourth, multivariate multinomial logistic regression analyses were performed to study the association between borderline personality symptoms with work performance. Work performance was categorized as having 0, 0.1–5 or > 5.1 days of work loss, and the reference category in these analyses consisted of those who reported 0 work loss days in the past month. Again, in the first series of analyses, adjustments were made for sex and age. In the second series of analyses, additional adjustments were made for education, living situation, any 12-month mood disorder, any 12-month anxiety disorder, and any 12-month substance use disorder. In the third series of analyses, the association of borderline personality symptoms and work performance was additionally adjusted for type or number of adverse working conditions to study the association between borderline personality symptoms and work performance mediated by type or number of adverse working conditions. Two-tailed testing procedures were used with 0.05 alpha levels in all analyses.

## Results

### Number of borderline personality symptoms

In this community-based sample of 3672 working people, 72.8% had no symptoms of borderline personality, 23.8% had 1–2 symptoms, 2.7% had 3–4 symptoms, and 0.7% had ≥5 symptoms (mean 0.45 (SE = 0.02)) (not in table). Younger age, lower education, living without a partner and the co-occurrence of any CMD were significantly associated with a higher number of borderline personality symptoms (Table 1).

**Table 1:** Sociodemographic characteristics among workers with borderline personality symptoms (*N* = 3672)

	Total working population		Borderline personality symptoms (0–6)	*P*-value
	n	%	Mean	
Total	3672	100	0.45	
Sex				0.27
Male	1831	56.4	0.43	
Female	1841	43.6	0.48	
Age				**0.002**
21–37	999	36.0	0.52	
38–47	1187	29.2	0.45	
48–57	1033	25.6	0.40	
58–64	453	9.2	0.34	
Education				**< 0.0001**
Lower secondary	859	24.1	0.58	
Higher secondary	1272	42.7	0.44	
Higher professional/ University	1541	33.2	0.37	
Living situation				**< 0.0001**
With partner	2676	71.9	0.40	
Without partner	996	28.1	0.59	
Any 12-month common mental disorder				
Mood disorder				
No mood disorder	3516	95.4	0.40	**< 0.0001**
Any mood disorder	156	4.6	1.59	
Anxiety disorder				
No anxiety disorder	3486	94.0	0.41	**< 0.0001**
Any anxiety disorder	186	6.0	1.19	
Substance use disorder				
No substance use disorder	3565	96.0	0.42	**0.001**
Any substance use disorder	107	4.0	1.19	

Significant associations highlighted in bold

### Working conditions

The adjusted associations between borderline personality symptoms and working conditions are summarized in Table 2. Borderline personality symptoms were associated with less decision latitude, less job security and less co-worker support. These associations persisted after adjustment for sociodemographic characteristics and CMD’s (Table 2, Model 2). Higher number of borderline personality symptoms was incrementally associated with poorer job quality, indicated by a higher number of adverse working conditions. The strength of these associations attenuated slightly in the model incorporating all covariates (Table 2, Model 2).

**Table 2:** Borderline personality symptoms as a correlate of working conditions among workers (*N* = 3672)

Borderline personality symptoms				
	n	mean	Adj. coefficient [95% CI] Model 1	Adj. coefficient [95% CI] Model 2
Type of working condition				
Decision latitude (24–96)	3661	74.25	**−1.26 [− 1.75- -0.76]**	**− 0.75 [− 1.26- -0.25]**
Psychological job demands (12–48)	3657	30.43	0.13 [− 0.12–0.37]	0.19 [− 0.08–0.45]
Job security (3–10)	3635	8.54	**− 0.18 [− 0.25- -0.12]**	**−0.15 [− 0.22- -0.08]**
Co-worker support (4–16)	3338	12.33	**−0.09 [− 0.16- -0.02]**	**−0.07 [− 0.14- -0.002]**
Number of adverse working conditions	n	%	Adj. RRR [95% CI] Model 1	Adj. RRR [95% CI] Model 2
0 (optimal)	1487	40.5	Ref	Ref
1	1394	38.2	**1.15 [1.02–1.29]**	1.08 [0.96–1.21]
2	572	16.3	**1.39 [1.21–1.59]**	**1.29 [1.11–1.49]**
3 or more	179	5.0	**1.64 [1.41–1.90]**	**1.41 [1.19–1.66]**

*Adj* Adjusted, *CI* Confidence interval, *RRR* Relative Risk Ratios

Ref: Reference category (no adverse working conditions)

Model 1: Adjusted for sex and age

Model 2: Adjusted for sex, age, education, living situation, any 12-month mood disorder, any 12-month anxiety disorder, any 12-month substance use disorder

Significant associations highlighted in bold

### Work performance

Borderline personality symptoms among workers were associated with impaired work performance, assessed in total work loss days. The mean of total work loss days was 2.0 (SE = 0.1) (not in table). The number of borderline personality symptoms was consistently associated with impaired work performance, in both categories of work loss (0.1–5 and > 5.1 days), also after adjustment for sociodemographic characteristics, CMD and type or number of adverse working conditions (Tables 3 and 4, Model 3).

**Table 3:** Borderline personality symptoms among workers (*N* = 3672) and type of working conditions as correlates of impaired work performance

Work loss days	0 days	0.1–5 days			> 5.1 days		
		Model 1 Adj. RRR [95% CI]	Model 2 Adj. RRR [95% CI]	Model 3 Adj. RRR [95% CI]	Model 1 Adj. RRR [95% CI]	Model 2 Adj. RRR [95% CI]	Model 3 Adj. RRR [95% CI]
Borderline personality symptoms (0–6)	Ref	**1.25 [1.13–1.38]**	**1.20 [1.08–1.34]**	**1.14 [1.00–1.28]**	**1.36 [1.22–1.51]**	**1.21 [1.07–1.37]**	**1.16 [1.02–1.33]**
Type of working condition							
Decision latitude (24–96)	Ref	1.00 [0.99–1.01]	0.99 [0.98–1.01]	1.00 [0.99–1.01]	**0.98 [0.97–1.00]**	**0.99 [0.97–1.00]**	0.99 [0.98–1.00]
Psychological job demands (12–48)	Ref	1.00 [0.98–1.03]	1.00 [0.98–1.02]	1.00 [0.97–1.02]	**1.04 [1.01–1.06]**	**1.04 [1.01–1.06]**	**1.03 [1.00–1.06]**
Job security (3–10)	Ref	**0.85 [0.78–0.92]**	**0.86 [0.80–0.94]**	**0.86 [0.79–0.93]**	**0.83 [0.76–0.91]**	**0.85 [0.78–0.94]**	**0.88 [0.80–0.97]**
Co-worker support (4–16)	Ref	0.97 [0.91–1.03]	0.96 [0.90–1.02]	0.98 [0.92–1.05]	0.95 [0.88–1.03]	0.96 [0.88–1.05]	1.00 [0.92–1.10]

*Adj* Adjusted, *CI* Confidence interval, *RRR* Relative Risk Ratios

Ref: Reference category (0 days of work loss)

Model 1: adjusted for demographic variables sex and age,

Model 2: adjusted for sex, age, living situation, education and any 12-month mood disorder, any 12-month anxiety disorder and any 12-month substance use disorder,

Model 3: adjusted for model 2 as well as all variables in the column (borderline personality symptoms and the four working conditions).

Significant associations highlighted in bold

**Table 4:** Borderline personality symptoms among workers (*N* = 3672) and number of adverse working conditions as correlates of impaired work performance

Work loss days	0 days	0.1–5 days			> 5.1 days		
		Model 1 Adj. RRR [95% CI]	Model 2 Adj. RRR [95% CI]	Model 3 Adj. RRR [95% CI]	Model 1 Adj. RRR [95% CI]	Model 2 Adj. RRR [95% CI]	Model 3 Adj. RRR [95% CI]
Borderline personality symptoms (0–6)	Ref	**1.25 [1.13–1.38]**	**1.20 [1.08–1.34]**	**1.17 [1.04–1.31]**	**1.36 [1.22–1.51]**	**1.21 [1.07–1.37]**	**1.19 [1.04–1.35]**
Number of adverse working conditions							
0 (optimal)							
	Ref	Ref	Ref	Ref	Ref	Ref	Ref
1	Ref	1.26 [0.95–1.68]	1.21 [0.89–1.63]	1.20 [0.88–1.63]	1.29 [0.99–1.70]	1.23 [0.93–1.63]	1.22 [0.92–1.63]
2	Ref	1.41 [0.97–2.04]	1.43 [0.97–2.11]	1.39 [0.94–2.05]	**1.69 [1.16–2.46]**	**1.54 [1.07–2.23]**	**1.49 [1.04–2.15]**
3 or more	Ref	**2.68 [1.55–4.65]**	**2.49 [1.48–4.18]**	**2.38 [1.41–4.01]**	**2.64 [1.64–4.26]**	**2.21 [1.36–3.60]**	**2.11 [1.30–3.43]**

*Adj* Adjusted, *CI* Confidence interval, *RRR* Relative Risk Ratios

Ref: Reference category (0 days of work loss) in the multinomial analyses and in the row (0 adverse working conditions)

Model 1: adjusted for demographic variables sex and age,

Model 2: adjusted for sex, age, living situation, education and any 12-month mood disorder, any 12-month anxiety disorder and any 12-month substance use disorder,

Model 3: adjusted for model 2 as well as all variables in the column (borderline personality symptoms and the four working conditions).

Significant associations highlighted in bold

In the model that included both borderline personality symptoms and each of adverse working conditions separately (Table 3, Model 3), we found that job insecurity was significantly associated with 0.1–5 work loss days compared to 0 work loss days. Decision latitude, psychological job demands and job security were significantly associated with > 5 work loss days compared to 0 work loss days, after controlling for sociodemographic characteristics and CMD (Table 3). After additionally controlling for the other types of working conditions and borderline personality symptoms (Table 3, Model 3), the significant association with decision latitude disappeared. Those reporting 3 or more adverse working conditions had higher risk of impaired work performance compared to workers with no adverse working conditions, decreasing slightly per model incorporating more covariates (Table 4, Models 2 and Models 3). Furthermore, in all models the number of borderline personality symptoms was significantly associated with impaired work performance, independent of type or number of adverse working conditions and any concurrent CMD.

## Discussion

To our knowledge, this is the first study examining the interplay between borderline personality symptoms and working conditions as a pathway for work performance in a general population sample. Although the actual number of people with fully developed BPD in the general population is relatively small (in this sample 0.7%), the present study shows that lower number of borderline personality symptoms are both common and associated with impaired work performance, independent of the type or number of adverse working conditions and concurrent CMD. After adjustment for CMD, the number of borderline personality symptoms was significantly associated with low decision latitude, job insecurity and low co-worker support, however not with psychological job demands.

The low rate of respondents with ≥5 symptoms of borderline personality might be explained by the association between BPD and unemployment or long-term disability benefits [1, 4]. Furthermore, our findings are based on an epidemiological working population which potentially differs from a clinical population. From this it is conceivable that those with fully developed BPD are more likely to be unemployed than employed [5]. This low prevalence might lead to an underestimation of the contribution of ≥5 borderline personality symptoms to working conditions. We hypothesized that the effect of borderline personality symptoms could contribute to adverse working conditions. As expected, the number of borderline personality symptoms was associated with decision latitude, even after adjustment for CMD. The relation with decision latitude could be explained by difficulties in decision-making and controlling of impulses in persons with BPD [13, 14], which may result in feelings of low control.

Contrary to our hypothesis, the association between borderline personality symptoms and psychological job demands was not significant. Despite the association between BPD and higher stress levels both in employment [31] and in general, showing more intense states of aversive tension compared to healthy controls [32]. However, the relation showed an expected increase of psychological job demands, this was not significant.

As anticipated, the number of borderline personality symptoms was associated with job insecurity. Individuals with borderline personality symptoms are associated with dismissal and demotion [2, 3], which possibly increases the fear of losing a job. Furthermore, data collection took place during times of economic crises, which naturally increases job insecurity. Nevertheless, it is still conceivable that job insecurity also contributes to deterioration of mental health [17].

As expected, borderline personality symptoms were negatively related to co-worker support. Interpersonal problems, which are a core symptom of BPD, are likely to arise as conflicts at work [2, 15, 31]. Individuals with borderline personality symptoms are less capable of reporting accurately on their experiences or on the effect of their behaviour upon others [3, 33]. Moreover, it is conceivable that individuals with borderline personality symptoms underestimate the effect of their behaviour, which can lead to conflicts and less co-worker support. However, the JCQ questions are fairly straightforward and minimise the potential of inaccurately reporting on this working condition.

We found that borderline personality symptoms were associated with impaired work performance, regardless of (adverse) working conditions and concurrent CMD. Our study confirms previous findings that psychopathology is associated with impaired work performance [7] and that higher number of adversities contribute to a deterioration of work performance [18]. Previous studies have shown that BPD is associated with unemployment and long-term disability benefits [1, 4]. Rehabilitation programs to increase skills for those in unemployment might be difficult due to a lack of social context. However, we have studied work performance in workers with borderline personality symptoms still being employed. Our findings show that even workers with few borderline personality symptoms demonstrate impaired work performance. This suggests that programs aimed at increasing work performance might be beneficial for those workers. Furthermore, detecting workers with borderline personality symptoms and increasing their skills in the workplace might prevent from potential long-term unemployment. However, as this is the first study that simultaneously evaluates (adverse) working conditions and borderline personality symptoms on work performance, comparison with other studies was not possible.

Using a population-based approach allowed us to study the associations between borderline personality symptoms and work performance with less risk of selection bias and a greater generalizability than clinical studies. Nevertheless, a number of limitations must be considered. Symptoms of borderline personality were measured with eight questions from the IPDE. Despite evidence that the IPDE was found valid for assessing BPD without the suicidality criterion, this is a limitation since the IPDE is unsuitable for the assessment of BPD in clinical practice. However, the IPDE can be used in epidemiological studies aimed at prevalence and associated correlates [5]. Furthermore, our findings are cross-sectional and, therefore, it is impossible to draw any causal relationships. Although the idea that borderline personality symptoms contribute to adverse working conditions and subsequently impair work performance is plausible, it is also possible that adverse working conditions contribute to an increase in traits, as has previously been shown for CMD [18, 27, 28]. Future studies should address borderline personality symptoms in work performance longitudinally. We were able to test a number of working conditions, however other domains of working conditions may be relevant which we were unable to study. Examples are downsizing in companies, procedural and organizational injustice, exposure to (sexual) violence and threats and role conflicts [34].

## Conclusions

Longitudinal studies are warranted to study the causal relationships between borderline personality symptomatology, working conditions and work performance. The present findings suggest that future studies should examine interventions aimed at diminishing borderline personality symptoms and coping with or changing of working conditions to subsequently reduce impaired work performance. Also, those still in employment are more likely to increase their skills while being in a social context. As previously shown [5, 35], even workers with low numbers of borderline personality symptoms were associated with impaired functioning. This suggests that treatment and research should focus on the broad spectrum of BPD, from lower to higher number of symptoms, both in and out employment.

## Abbreviations

BPD: Borderline personality disorder
CMD: Common mental disorders
DSM: Diagnostic and Statistical Manual of Mental Disorders
